# Astral architecture can enhance mechanical strength of cytoskeletal networks by modulating percolation thresholds

**DOI:** 10.1016/j.bpj.2026.03.028

**Published:** 2026-03-31

**Authors:** Brady Berg, Jun Allard

**Affiliations:** 1 Mathematical, Computational and Systems Biology, University of California, Irvine, Irvine, California; 2 Department of Mathematics, Department of Physics & Astronomy, Center for Complex Biological Systems, University of California, Irvine, Irvine, California

## Abstract

A repeated pattern in cytoskeletal architecture is the aster, in which a number of F-actin filaments emerge star shaped from a central node. Aster-based structures occur in cytoplasmic actin, the early stages of the cytokinetic ring in yeast, and in the context of biomimetic materials engineering. In this work, we use computational simulation to show that there is an optimal number of filaments per aster that maximizes rigidity, even at a fixed density of F-actin. This nonlinear dependence holds for both the shear and extensional moduli. At physiological parameters, the maximum corresponds approximately to the same filaments per aster observed in recent super-resolution images of cortical F-actin. Furthermore, we find that increasing filaments per aster leads to dramatic increases in the sample-to-sample variability in network rigidity. We explain both effects using percolation theory, wherein the probability that a given network is productively connected exhibits a sharp dependence on parameters. The dependence of network rigidity on this nanoscale architectural feature may suggest a mechanism by which cells tune the physical properties of their actin networks locally and rapidly (since no new F-actin must be assembled) and may inform efforts to create adaptive synthetic metamaterials inspired by actin networks.

## Significance

Arranging the components of a structure in different ways can alter its mechanical strength, even in the presence of overall disorder. In particular, star-shaped aster arrangements appear in many parts of the cell cytoskeleton. We use computational simulation to study the mechanical strength of flexible filaments arranged in different aster configurations. We find that asters increase mechanical strength up to a point, after which they lead to too many gaps in the structure. Interestingly, the optimal aster arrangement matches previous observations using microscopy. Aster formation also aligns with measurements of how cells dynamically tune their cytoskeleton. Aster structures could provide a tunable, optimizable arrangement in dynamic cells and inspire novel biomimetic metamaterials.

## Introduction

As the key component of cell mechanical structures, the mechanical properties of F-actin are crucial to understanding cell biology (1,2). Much progress has been achieved understanding F-actin at the scale of individual filaments (3,4), and at the scale of cells, where F-actin forms a cytoskeleton that can be understood as an active gel using continuum models (5). Connecting those two scales—the nanoscale architecture—has proven more challenging. Nanoscale architecture has been studied in physical theory and computational simulations (6,7,8,9,10,11,12,13,14). With the advent of super-resolution microscopy, an increasing number of nanoscale architectures have been experimentally studied, for example the low-density cytosolic network (15,16) and the cortex (17). Many of these structures are approximately two-dimensional, for example, the cortex (18,19,20) and the cytokinetic ring (21).

Several recent works have demonstrated that classical percolation theory is useful in understanding the nanoscale architecture of the actin cytoskeleton and other filamentous structures (6,22,23,24,25,26,27). This theory is based on the phenomenon that, as network density is decreased, there is a critical density below which the filaments no longer make a connected set and are therefore not able to sustain mechanical resistance.

**Figure 1:**
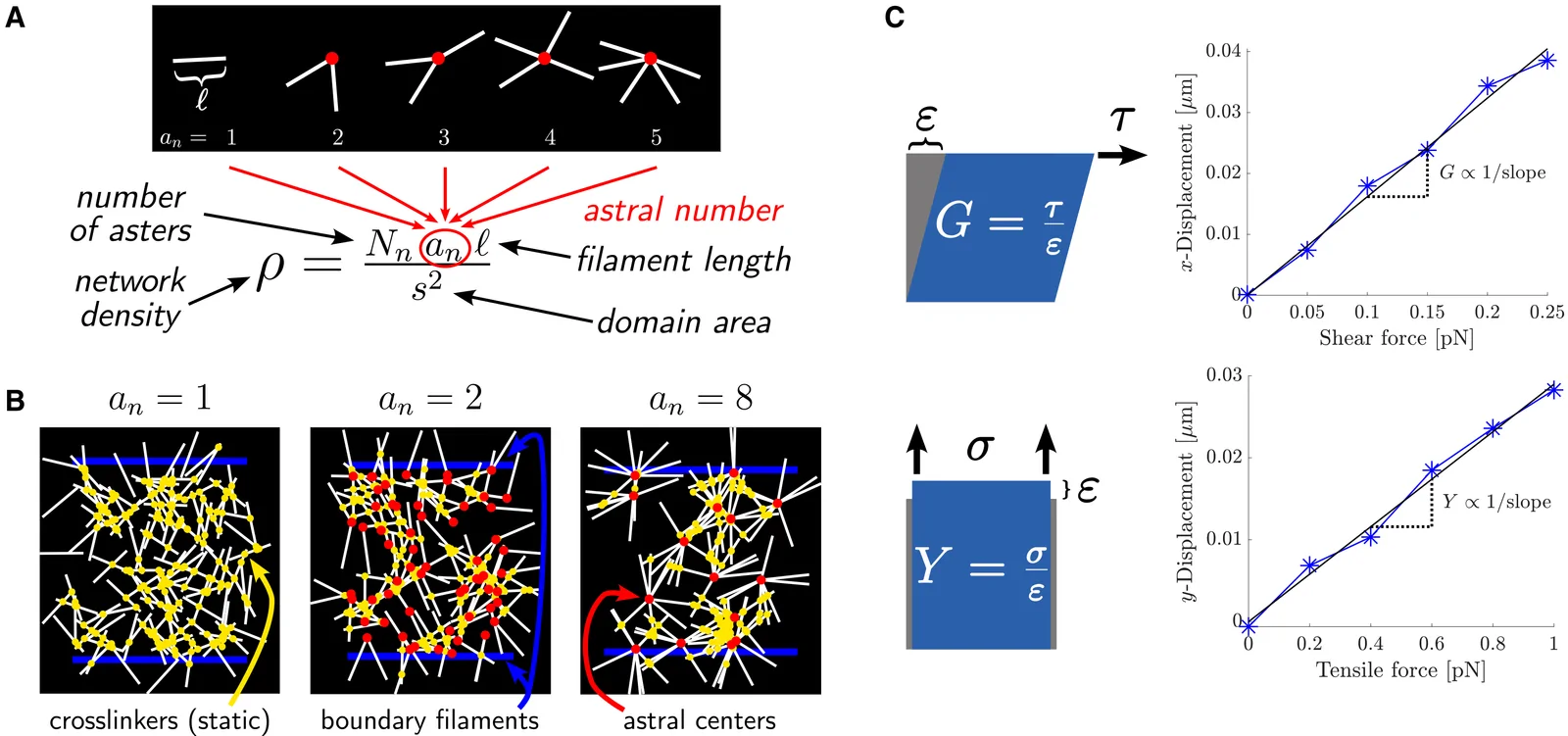
Framework for studying astral network rigidity. (*a*) Schematic of asters and their astral number *a*_*n*_, i.e., filaments per aster. Variations in *a*_*n*_ at a fixed network density *ρ* correspond to changes in the nanoscale arrangement of a set number of filaments. (*b*) Schematics of filament networks at fixed density *ρ* and astral numbers 1 (*left*), 2 (*middle*), and 8 (*right*). Cross-linkers are shown in yellow, boundary filaments are shown in blue, and astral centers are shown in red. (*c*) Schematics of bulk shear modulus *G* (*top*) and bulk Young’s modulus *Y* (*bottom*). We estimate these elastic moduli from the slope of displacement (proportional to strain) versus force (proportional to stress) simulation data (see materials and methods for details).

A recurring observation in vivo (15,16,17), in vitro (28), and in computational simulations (28,29) is the emergence of asters, in which several actin filaments emanate from an astral center in a star-like pattern, as shown in Fig. 1. Kruse et al. (30) provide evidence that asters are a ubiquitous, fundamental, elementary unit of cellular organization. Actin asters are varied in structure, with centers containing formin (15) or Arp2/3 (17), either requiring myosin (28) or not (17). In vivo, the precise quantification of asters remains challenging, but recently, Xia et al. (17) used super-resolution microscopy to find that the cortex of embryonic stem cells was composed of asters with *a*_*n*_ ≈ 3 − 6 filaments per aster, what they term the “astral coordination number” and we herein call “astral number” *a*_*n*_. Prior work has queried how asters may emerge (28,29). Here instead, we ask, what are the biophysical consequences of asters?

In this work, we simulate a minimal computational model of astral polymer networks and measure their mechanical strength. The model takes as input the formation of asters with a particular astral number, linked with additional interaster cross-links. We find an optimal astral number that maximizes the mechanical moduli, for a fixed total amount of polymer. Furthermore, the astral number that maximizes mechanical strength is in close agreement with the aster coordination number of (17), suggesting that the cells under their study were optimizing nanoscale architecture to maximize strength for a given amount of F-actin. We find that the optimum can be explained by percolation theory, specifically the modulation of critical percolation thresholds. In addition, the results qualitatively explain a previously reported nonlinear phase diagram of in vivo F-actin (27).

## Materials and methods

### Mechanical simulations

Mechanical simulations of astral cytoskeletal networks were carried out using a custom extension of Cytosim (31). Filaments were modeled as inextensible filaments of length *ℓ* = 0.1 μm and bending rigidity 0.01 pN μm^2^. Asters group filaments into radial assemblies via the action of two springs at the astral center with stiffnesses 500 pN μm^−1^ and 250 pN μm^−1^. The former pins one end of a filament to the astral center, and the latter resists a filament’s rotation about the astral center. Unless otherwise indicated, orientations of filaments about each astral center were initialized uniformly at random.

Each astral network consists of a number of asters distributed uniformly at random within a square domain of side length *s* = 1 μm. There is no volume-exclusion effect in the simulation, since this is a two-dimensional approximation of a three-dimensional system where filaments can cross out of plane. All asters in a particular network have the same number of filaments per central node, and we use the term “astral number,” *a*_*n*_, to refer to this quantity (see also Fig. 1 *a*). In addition to asters, two additional boundary filaments were placed at the top and bottom of the domain. Each boundary filament has length equal to *s*, the domain size, and rigidities equal to 500 pN μm^2^.

Irreversibly binding cross-linkers were attached to random points along network filaments at time *t* = 0 s. Cross-linkers have binding radii of 0.001 μm^−1^ and (linear) stiffnesses of 50 pN μm^−1^. Cross-linkers do not individually exert torques. Unless otherwise indicated, the number of cross-linkers used was 30 × (number of astral filaments) + 100 × (number of boundary filaments), which at density 75 μm^−1^ corresponds to 22.7 × 10^3^ particles μm^−2^. A high density of cross-linkers was selected to encourage binding between filaments before they have time to diffuse away from their initial positions, thus mimicking the simulations of (6) where cross-links were placed wherever filaments intersect. Network components are schematized in Fig. 1 *b*. See Table 1 for a summary of the key biophysical parameters used in Cytosim; unless otherwise stated, parameters assume the indicated default value. Parameters related to the numerical scheme are summarized in Table 2.

**Table 1:** Biophysical Parameters

Parameter (Symbol)	Default value	Description
System size (*s*)	1 μm	side length of square domain
Network density (*ρ*)	75 μm^−1^	average filament length per unit area
Filament length (*ℓ*)	0.1 μm	inextensible; no growth/disassembly/fracture
Filament bending rigidity	0.01 pN μm^2^	–
Thermal energy scale (*k*_*B*_*T*)	2.1 × 10^−5^ pN μm (reduced *k*_*B*_*T*); 4.2 × 10^−3^ pN μm (physiological *k*_*B*_*T*)	–
Astral center stiffness #1	500 pN μm^−1^	attaches filament ends to center of aster
Astral center stiffness #2	250 pN μm^−1^	resists filament rotation about center of aster; acts 0.03 μm from center
Boundary filament bending rigidity	500 pN μm^2^	–
Boundary anchor stiffness	500 pN μm^−1^	fix lower boundary filament in place (and upper boundary during cross-linking phase)
Boundary anchor spacing	0.1 μm	anchors placed along lower boundary filament
Cross-linker concentration	22.7×10^3^ particles μm^−2^	see materials and methods for details
Cross-linker stiffness	50 pN μm^−1^	–
Cross-linker binding rate	1 s^−1^	–
Cross-linker unbinding rate	0	cross-linkers do not unbind
Cross-linker binding range	0.001 μm	–
Shear force range	0 pN–0.25 pN (reduced *k*_*B*_*T*); 0 pN–1.25 pN (physiological *k*_*B*_*T*)	six equally spaced forces per network
Tensile force range	0 pN–1 pN (reduced *k*_*B*_*T*); 0 pN–5 pN (physiological *k*_*B*_*T*)	six equally spaced forces per network
Viscosity	0.5 pN s/μm^2^	–

**Table 2:** Cytosim simulation parameters

Parameter	Default Value	Description
Time step	0.1 s	–
Numerical tolerance	0.05	default value, unitless
Filament segmentation	0.02 μm	length of rigid filament sub-segments
Boundary filament segmentation	0.05 μm	as above but for boundary filaments
Duration of cross-linking phase	50 s	system evolves without any applied force; initial network position recorded at end of this phase
Duration of force application phase	250 s	constant applied force; final network position recorded at end of this phase

Each simulation begins by letting network components (asters with attached cross-linkers) diffuse freely for 50 s, allowing cross-linkers to bind asters together into a particular network geometry. During this time, the top and bottom boundary filaments are held in place. Since cross-linkers do not unbind, the network geometry that forms is dictated primarily by the random initialization of aster positions.

To study the response of each network to an applied force, we customized Cytosim to enable the application of force to the ends of particular network fibers. For reduced *k*_*B*_*T* simulations, the position of each fiber in the network was recorded at *t* = 50 s and used as the initial state of the network. For physiological thermal energy levels, 10 position samples were collected from equally spaced time points in the interval *t* = 41*–*50 s and the average of these positions was used as the initial state of the network. Also at *t* = 50 s, we recorded which filaments were cross-linked together. Simultaneously, the anchors attached to the top boundary filament were removed, and a constant force on this filament was switched on. For shear force simulations, a rightward force was applied to the right-hand end of the top boundary filament. For tensile force simulations, upward forces were applied to each of the left- and right-hand ends of the top boundary filament, each with magnitude half of the reported applied force. Although the bottom boundary filament remained pinned, the network was allowed to deform until *t* = 300 s, by which time networks achieve steady state (Figs. S3 *a*, *c*, S1 *a*, and *c*). For reduced *k*_*B*_*T* simulations, the position of each fiber in the network was recorded at *t* = 300 s and used as the final state of the network. For physiological thermal energy levels, 10 position samples were collected from equally spaced time points in the interval *t* = 291*–*300 s, and the average of these positions was used as the initial state of the network. Center-of-mass network displacements were computed by averaging the positions of all network fibers (including an average over each sampled time point, if appropriate) and subtracting final position from initial position. Boundary filaments were excluded from center-of-mass computations. For shear, we used the horizontal (*x*) component of displacement to estimate an elastic modulus, and in extension, we used the vertical (*y*) component.

For representative simulations at reduced *k*_*B*_*T* parameters, see Videos S9, S10, S11, and S12 (shear force) and Videos S13, S14, S15, and S16 (tensile force). Analogous simulations at physiological thermal energy levels are given in Videos S1, S2, S3, and S4 (shear force) and Videos S5, S6, S7, and S8 (tensile force). Center-of-mass positions of these networks over time are plotted in Fig. S3 *a* and *c* (reduced *k*_*B*_*T*) and Fig. S1 *a* and *c* (physiological *k*_*B*_*T*).

Video S1. Shear force (1.25 pN) applied to filament network with astral number 1 (Mikado network) at physiological temperature

Video S2. Shear force (1.25 pN) applied to astral filament network with astral number 4 at physiological temperature

Video S3. Shear force (1.25 pN) applied to astral filament network with astral number 8 at physiological temperature

Video S4. Shear force (1.25 pN) applied to astral filament network with astral number 16 at physiological temperature

Video S5. Tensile force (5 pN) applied to filament network with astral number 1 (Mikado network) at physiological temperature

Video S6. Tensile force (5 pN) applied to astral filament network with astral number 4 at physiological temperature

Video S7. Tensile force (5 pN) applied to astral filament network with astral number 8 at physiological temperature

Video S8. Tensile force (5 pN) applied to astral filament network with astral number 16 at physiological temperature

Video S9. Shear force (0.25 pN) applied to filament network with astral number 1 (Mikado network) at reduced thermal energy

Video S10. Shear force (0.25 pN) applied to astral filament network with astral number 4 at reduced thermal energy

Video S11. Shear force (0.25 pN) applied to astral filament network with astral number 8 at reduced thermal energy

Video S12. Shear force (0.25 pN) applied to astral filament network with astral number 16 at reduced thermal energy

Video S13. Tensile force (1 pN) applied to filament network with astral number 1 (Mikado network) at reduced thermal energy

Video S14. Tensile force (1 pN) applied to astral filament network with astral number 4 at reduced thermal energy

Video S15. Tensile force (1 pN) applied to astral filament network with astral number 8 at reduced thermal energy

Video S16. Tensile force (1 pN) applied to astral filament network with astral number 16 at reduced thermal energy

### Estimation of elastic moduli

To quantify network rigidity, we subjected each sampled network to a series of applied forces and computed the corresponding center-of-mass displacements (Fig. S3 *b* and *d* (reduced *k*_*B*_*T*), and Fig. S1 *b* and *d* (physiological *k*_*B*_*T*)). We generated *N* random seeds for Cytosim’s random number generator, corresponding to *N* unique initial geometries at each astral number. Specification of random seeds in the Cytosim config.cym file thus enabled us to apply multiple forces to the same network. For each network, we performed a sweep over six equally spaced force magnitudes from the ranges indicated in Table 1. We then constructed a displacement versus force curve and performed linear regression to estimate (the reciprocal of) an elastic modulus (Fig. 1 *c*). Fitting was done using the fit function from MATLAB R2024b (The MathWorks) with bisquare robust fitting enabled and intercept fixed to (0, 0). This fitting method is equivalent to estimating the slope of stress-strain data, since network stress is *F*_shear_/*s* (or *F*_tens_/*s*) and strain is Δ*x*/*s* (resp. Δ*y*/*s*). The elastic modulus assigned to each network is equal to the reciprocal of the slope of the linear fit described above, except it is set to 0 pN μm^−1^ if any of the following are observed.

1. Lack of a spanning component in the network as determined from the cross-linker data at *t* = 50 s
2. Changes in displacements between adjacent, nonzero applied forces of magnitude at least 75% of the maximum measured displacement for that network
3. (Only for reduced *k*_*B*_*T* simulations) A decrease between displacements in the zero applied force simulation and the lowest positive applied force simulation

**Figure 2:**
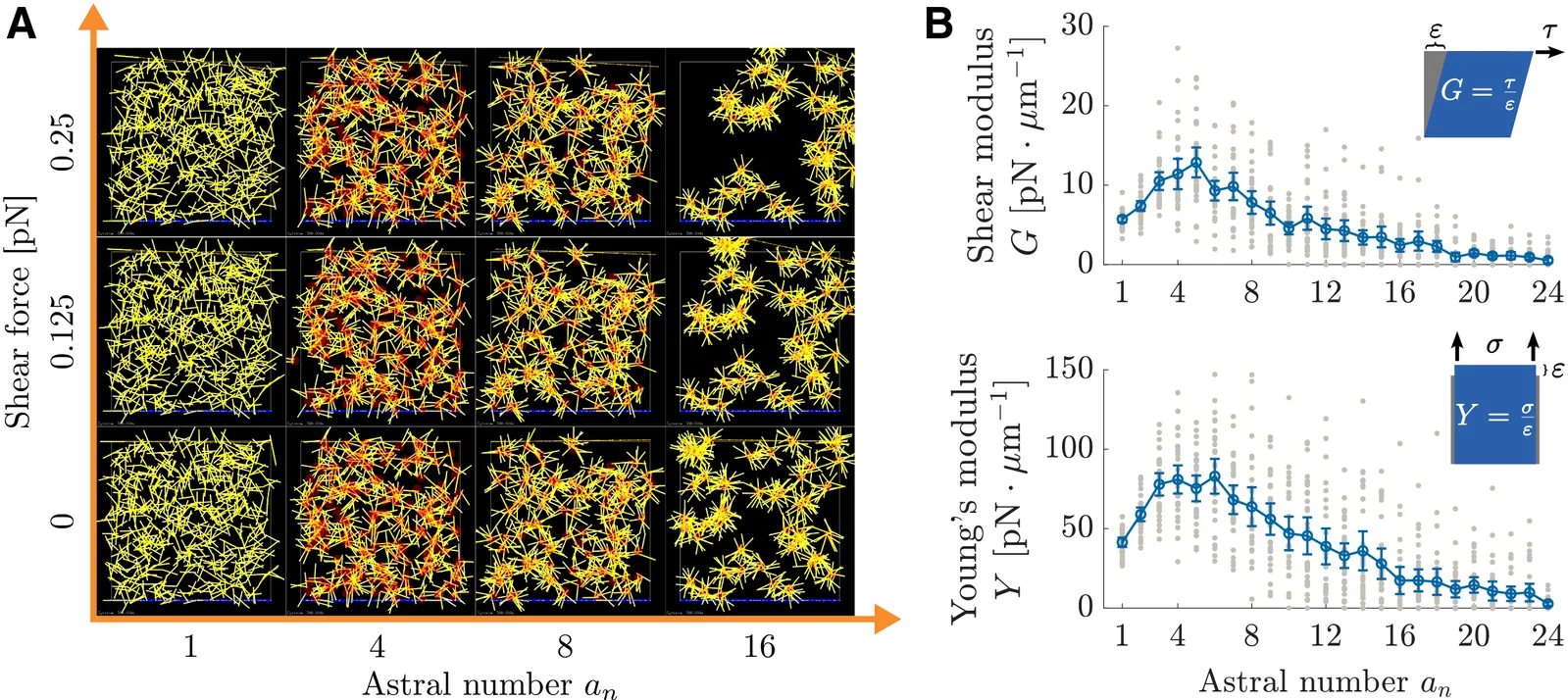
Rigidity of astral filamentous networks exhibits a maximum at intermediate astral number, even at fixed density. (*a*) Steady-state snapshots of astral network deformation in response to applied shear forces. All tiles have equal total number of filaments. (*b*) Elastic moduli of astral networks at density *ρ* = 75 μm^−1^ as a function of astral number. Upper: shear modulus; lower: Young’s (tensile) modulus. Plots show mean and 95% CIs from *N* = 30 network samples (see materials and methods for details). Individual network moduli are shown (*gray markers*).

We imposed a zero modulus in case 1 because a spanning component is necessary for a network to store energy from applied force. Condition 2 was imposed to catch networks that stochastically achieve a spanning component at certain force values but fail at others (e.g., oscillatory line in Fig. S3 *d* at *a*_*n*_ = 16). Condition 3 catches networks where a spanning component was detected from cross-linker data but nonetheless the network does not deform in the direction of the applied force (seven of 1440 networks shown in Fig. 2 *b*). Condition 3 was only used for reduced *k*_*B*_*T* simulations because, at physiological thermal energy levels, displacement magnitudes at the smallest positive force value were comparable to the scale of thermal fluctuations. In this case, condition 3 cannot be used to detect networks with faulty connectivity.

### Geometric analysis: Dangling ends and percolation

To study geometric properties of astral networks, we generated astral networks by distributing asters uniformly at random in a square domain and placing cross-links wherever filaments intersect. Generation and analysis code is written in MATLAB R2024b (The MathWorks). All measurements of dangling ends as well as counts of productive segments by node were conducted for networks with *ρ* = 75 μm^−1^, *ℓ* = 0.1 μm, and *s* = 1 μm. Network parameters for percolation probability estimates are provided in the relevant figure captions.

The static networks generated by this Monte Carlo code are an idealized approximation of the networks generated by Cytosim, which include thermal fluctuations and finite cross-linker dynamics. To measure the level of approximation, we compute percolation probabilities from the connectivity reported by cross-linker binding, shown in Fig. S9. Note this is fundamentally a different quantity than whether two lines cross each other, since we extract the connectivity from checking which two filaments each cross-linker is bound to. We suspected the main source of discrepancy is due to thermal fluctuations, the finite size of cross-linkers, and the stochastic binding and unbinding of cross-linkers in Cytosim. Indeed, increasing viscosity 2000-fold to 10^3^ pN/μm^2^ and decreasing cross-linker binding range 10-fold to 10^−4^ μm brings the two simulation approaches into agreement.

To analyze percolation in astral networks, we used the MATLAB function conncomp to identify sets of mutually cross-linked filaments, henceforth referred to as connected components. In the language of graph theory, each network filament is represented by a graph vertex, and an edge is drawn between vertices if those filaments were cross-linked together (i.e., if they intersected). A connected component was said to be “spanning” if it contained both a cross-link above and below the network boundaries (see Fig. 5 *a*), and an entire network was said to be spanning if it contained at least one spanning component. For “connectivity” percolation, we determined if a network had a unique connected component (see Fig. 5 *b*).

We define the critical percolation densities $\rho_{c}^{\text{span}}\left(a_{n}\right)$ and $\rho_{c}^{\text{conn}}\left(a_{n}\right)$ to be the network density at which the percolation probability of networks with astral number *a*_*n*_ equals 50%. To compute these values, we used the MATLAB function fit to compute smoothing spline fits (automatically selected smoothing parameter) to the percolation data. Using these fits, we numerically solved (using MATLAB’s fsolve, default tolerances) for the network density where the percolation probability reaches 50%. Example percolation data and fits are shown in Fig. S8.

## Results

### Computational model of astral filament network

To understand the properties of astral cytoskeletal networks, we extended the model of (6) to the case of astral network components. Asters are represented in the model as radial assemblies each with *a*_*n*_ filaments per central node (their astral number *a*_*n*_, see Fig. 1 *a*), each filament having fixed length *ℓ*. Every aster in a particular network has the same number of filaments per central node. To form a network, *N*_*n*_ asters were distributed in a square domain of area *s*^2^ and permanently binding cross-linkers were distributed to cross-link asters to each other and to the boundary at the top and bottom of the domain (Fig. 1 *b*). Coordinates of astral centers and the orientation of each filament about its respective center were sampled uniformly at random. We use network density *ρ* to refer to the average filament length per unit network area,

(1) $$\rho =\frac{a_{n}N_{n}\ell }{s^{2}}.$$

Unless otherwise specified, we use *ℓ* = 0.1 μm and *s* = 1 μm; for a complete list of default parameter values, see Table 1.

### Rigidity of astral filamentous networks exhibits a maximum at intermediate astral number

We next simulated networks for varying astral number *a*_*n*_ and network density *ρ* (Fig. S4 *a*). We quantified the rigidity of these astral networks by estimating an elastic modulus from the slope of stress-strain response curves. To do this, we applied a varying force to each sampled network, computed center-of-mass displacements at steady state, and estimated elastic modulus from the initial linear response (Fig. 1 *c*). Modulus estimation is detailed further in materials and methods. We investigated network behavior under shear and under tension. Representative images of networks experiencing a varying shear force are provided in Fig. 2 *a*.

We observed a peak in both the shear and Young’s (tensile) moduli at an intermediate astral number (Fig. 2 *b*). These peaks in modulus occur at similar astral numbers for both deformation modes. The astral number at which these peaks occur is insensitive to the specific network density tested (Fig. S4 *a*). Similarly, the position of the peak does not depend on cross-linker density or cross-linker stiffness (Fig. S5). The peak is not sensitive to the particular filament bending rigidity selected, nor to the presence or magnitude of angular stiffness (i.e., torque) at astral centers (Fig. S6).

**Figure 3:**
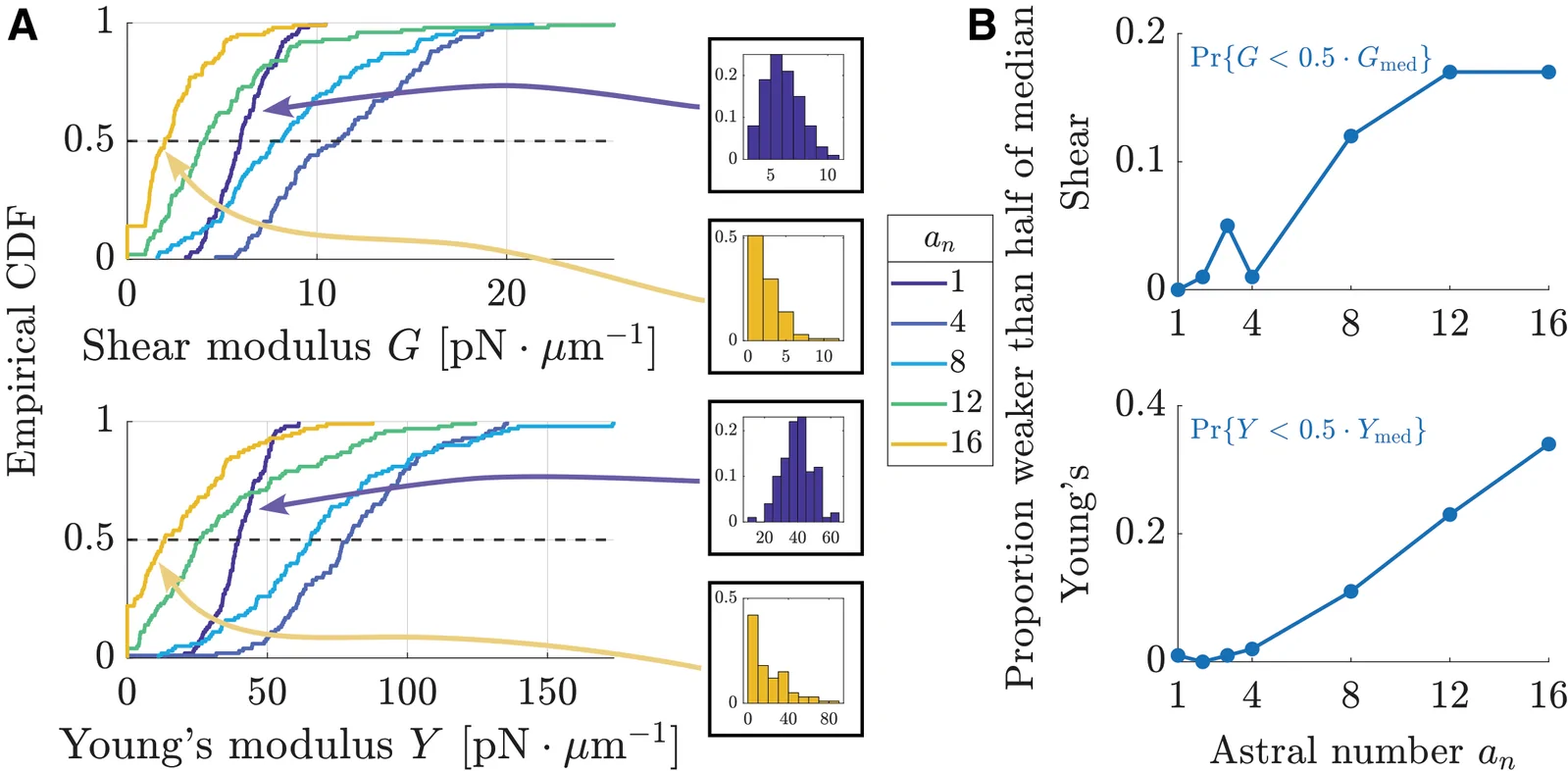
Weakening at high astral number is due to increasing probability of network failure. (*a*) Cumulative distribution functions and histograms for the elastic moduli of astral networks at *ρ* = 75 μm^−1^. Upper: shear modulus; lower: Young’s (tensile) modulus. (*b*) Proportion of astral networks that are weaker than half the median modulus. Distributions were estimated from *n* = 100 network samples (see materials and methods for details).

The mean behavior is also insensitive to the simulated temperature *k*_*B*_*T* (which determines the magnitude of random fluctuations), as shown in Fig. S2, although the time series are considerably more noisy at high *k*_*B*_*T* = 4pN nm, as can be seen by comparing Figs. S1 and S3. For this reason, when collecting statistics for Figs. 3, 5 *e* and 6 *c* below, we report results at reduced temperature *k*_*B*_*T* = 2.1 × 10^−2^ pN nm.

### Weakening at high astral number is due to increasing probability of network failure

The sample-to-sample variability in elastic modulus (bars in Fig. 2 *b*) also depends on astral number. Whereas for nonastral networks (*a*_*n*_ = 1) the variance in moduli is low, at moderate astral number, the spread of the sampled moduli values appears to increase. This suggests that network rigidity is more sensitive to the initial configuration of network components when astral number is higher. To investigate further the role of geometric variability in determining network rigidity, we estimated the distributions of the shear and Young’s moduli for various astral numbers (Fig. 3 *a*). As in Fig. 2 *b*, the distributions for shear and Young’s moduli have similar shapes, but this shape changes categorically depending on aster number. For nonastral networks (*a*_*n*_ = 1), the distributions of moduli have vanishing probability of very small moduli; i.e., their histograms sit away from the origin, and the cumulative distribution functions (CDFs) are flat at the origin (purple CDFs and histograms in Fig. 3 *a*), like a Gaussian distribution. Once astral number increases past *a*_*n*_ ≈ 8, the distributions become increasingly skewed toward low modulus values, losing the upward concavity at zero by *a*_*n*_ = 12 and showing a distribution mode near 0 by *a*_*n*_ = 16 (gold CDFs and histograms in Fig. 3 *a*), like an exponential distribution.

This marked shift from Gaussian-like to exponential-like distribution types suggests that increasing *a*_*n*_ beyond intermediate values results in a bias toward weak and mechanically incompetent networks at these astral numbers. We computed, at each sampled astral number, the proportion of networks that had modulus below half of the respective median modulus value (Fig. 3 *b*). For astral numbers below ≈4, the proportion of networks that are weak relative to the median is less than 5%. This quantity dramatically increases, approaching 20% (shear) and 30%−40% (Young’s) at high astral numbers, even as the median modulus at high astral numbers itself decreases (intersections with dashed lines in Fig. 3 *a*). Thus the weakening at high astral numbers is driven in part by an increase in the likelihood of a network geometry being incapable of withstanding mechanical load (e.g., due to fractures or gaps).

### Strength dependence on astral number is not associated with dangling filament fraction nor mean segments per node

**Figure 4:**
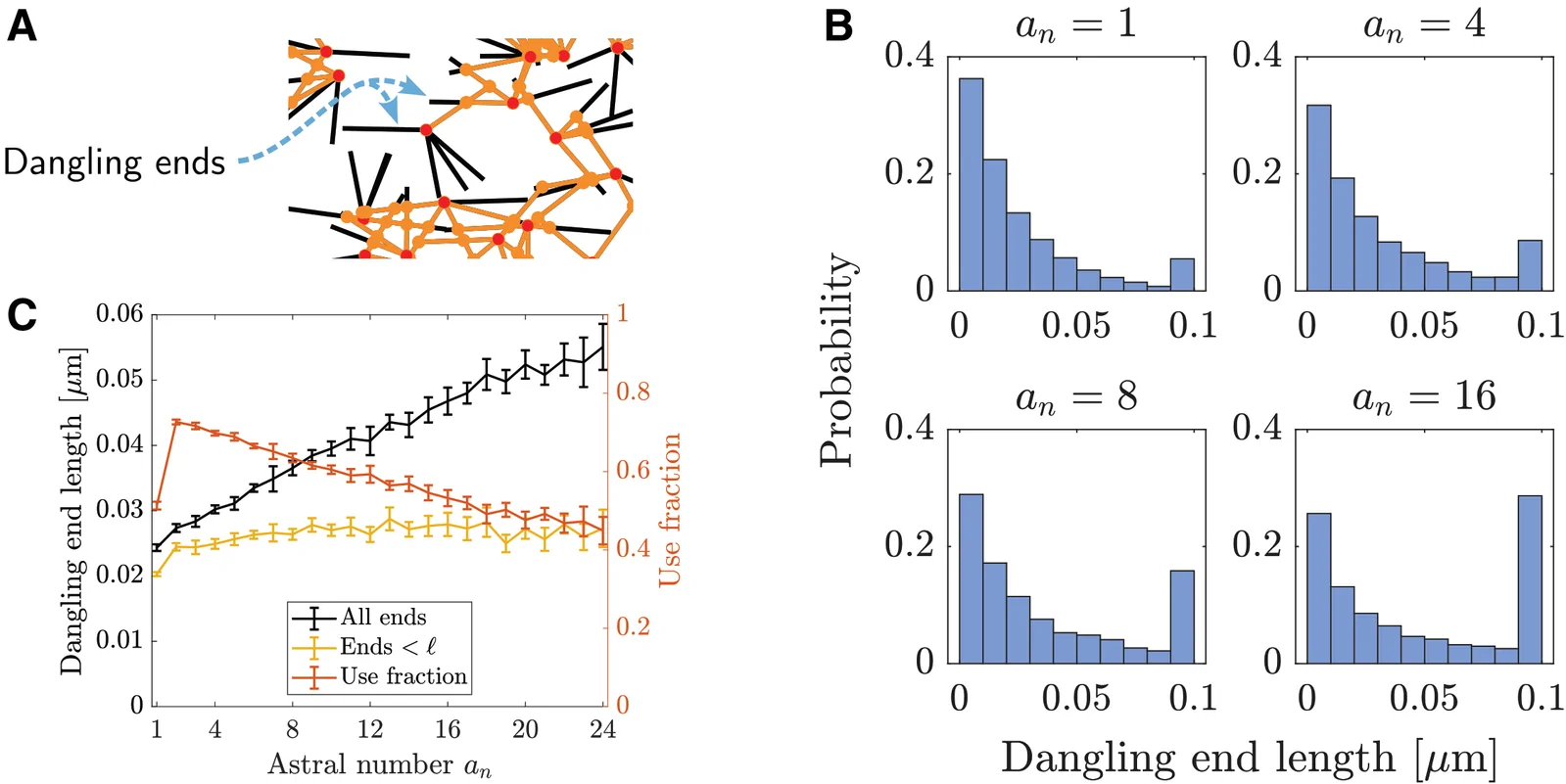
Strengthening over low astral numbers is not associated with dangling filament fraction. (*a*) Schematic of dangling ends (*black sections of filaments*) in astral networks. (*b*) Histograms of dangling end lengths at various astral numbers. Distributions are aggregated from the dangling ends in 10 networks at each astral number. (*c*) Mean dangling end length as a function of astral number. Also shown is the length “use-fraction,” defined in Eq. 2. Bars indicate 95% CIs estimated from 10 networks.

One a priori possible explanation for why networks with intermediate astral number are more rigid than nonastral networks is that the astral centers force more of the available filament length into mechanically productive structures. One way to classify if a filament segment is mechanically productive is to distinguish between filament segments that are bounded on both ends by a connection (either astral center, or interaster cross-link, shown as orange segments in Fig. 4 *a*) and those that are not; those in the latter category are thus dangling ends (black segments in Fig. 4 *a*). Dangling ends do not experience any net force when a network is deformed.

To test if changes in dangling ends could explain the rigidity increase shown in Fig. 2 *b*, we measured the dangling end lengths in networks with different astral numbers (see materials and methods for details). Distributions of dangling ends have the shape of an exponential distribution with a singular accumulation point corresponding to the maximum dangling end length *ℓ* = 0.1 μm (Fig. 4 *b*). At sufficiently high astral numbers, this accumulation point gathers enough probability that the distributions appear bimodal. The exponential part of these distributions is in agreement with analytic studies that predict the distribution of dangling ends to be exponential (32).

The mean dangling end length in astral networks increases monotonically with *a*_*n*_ (Fig. 4 *c*). This increase vanishes when inspecting only the dangling ends with lengths strictly below *ℓ*, showing that the change in mean dangling end length is driven by the number of totally isolated filaments. As a complimentary quantification, we computed the length “use-fraction” for all sampled networks, defined as

(2) $$\frac{\text{length}\;\text{not}\;\text{in}\;\text{dangling}\;\text{ends}}{\text{total}\;\text{filament}\;\text{length}\;\text{in}\;\text{network}}=1-\frac{\text{total}\;\text{dangling}\;\text{end}\;\text{length}}{\text{total}\;\text{filament}\;\text{length}\;\text{in}\;\text{network}}.$$

Although the use-fraction increases when *a*_*n*_ changes from 1 to 2, it decreases consistently for *a*_*n*_ ≥ 2. The initial increase in use-fraction is because there are fewer total dangling ends in astral networks, since filament ends located at astral centers are considered to be cross-linked to each other precisely at their ends. (In nonastral networks, both ends of a filament generate dangling ends of nonzero length.) Overall, the trends pertaining to dangling ends in astral networks does not agree with the trends in modulus in Fig. 2 *b*.

Classical rigidity analysis (33) suggests that the mean number of segments per node plays a role in determining deformability. Although the networks studied here are more complex (24,34), we compute the mean segments per node, and its full distribution, as a function of aster number, in Fig. S7. There are two kinds of nodes between filaments in this model: astral centers, which all have fixed *a*_*n*_ filaments each, and then interaster cross-link connections. The interaster cross-links connect either two, three, or four productive segments, where two line segments may belong to the same filament. We find that the mean number of segments (for both kinds of nodes) gradually increases from around 3.1 to around 3.9 as astral number increases from *a*_*n*_ = 1 to 24, without an optimum or crossing integer values.

### Mechanical rigidity is associated with network connectedness

Having confirmed that dangling ends and use-fraction do not explain the maximum in astral number, we next noted that at high astral numbers, filaments are highly concentrated around relatively few nodes, making it extremely unlikely that network components connect into a structure capable of resisting force. After this, we sought to study the connectivity properties of astral networks by quantifying their percolation thresholds, a geometric measure that has previously proven useful in understanding the actin cytoskeleton and other filamentous structures (6,22,23,24,25,26,27). In brief, the central idea of percolation theory is that as network density increases, the probability that a random network is connected transitions sharply from 0 to 1. The density at which this transition occurs is known as the percolation threshold. Though we restrict our attention to finite networks in this work, the behavior of finite systems is similar and approaches the infinite size for *s* ≫ *ℓ* (Fig. S8).

**Figure 5:**
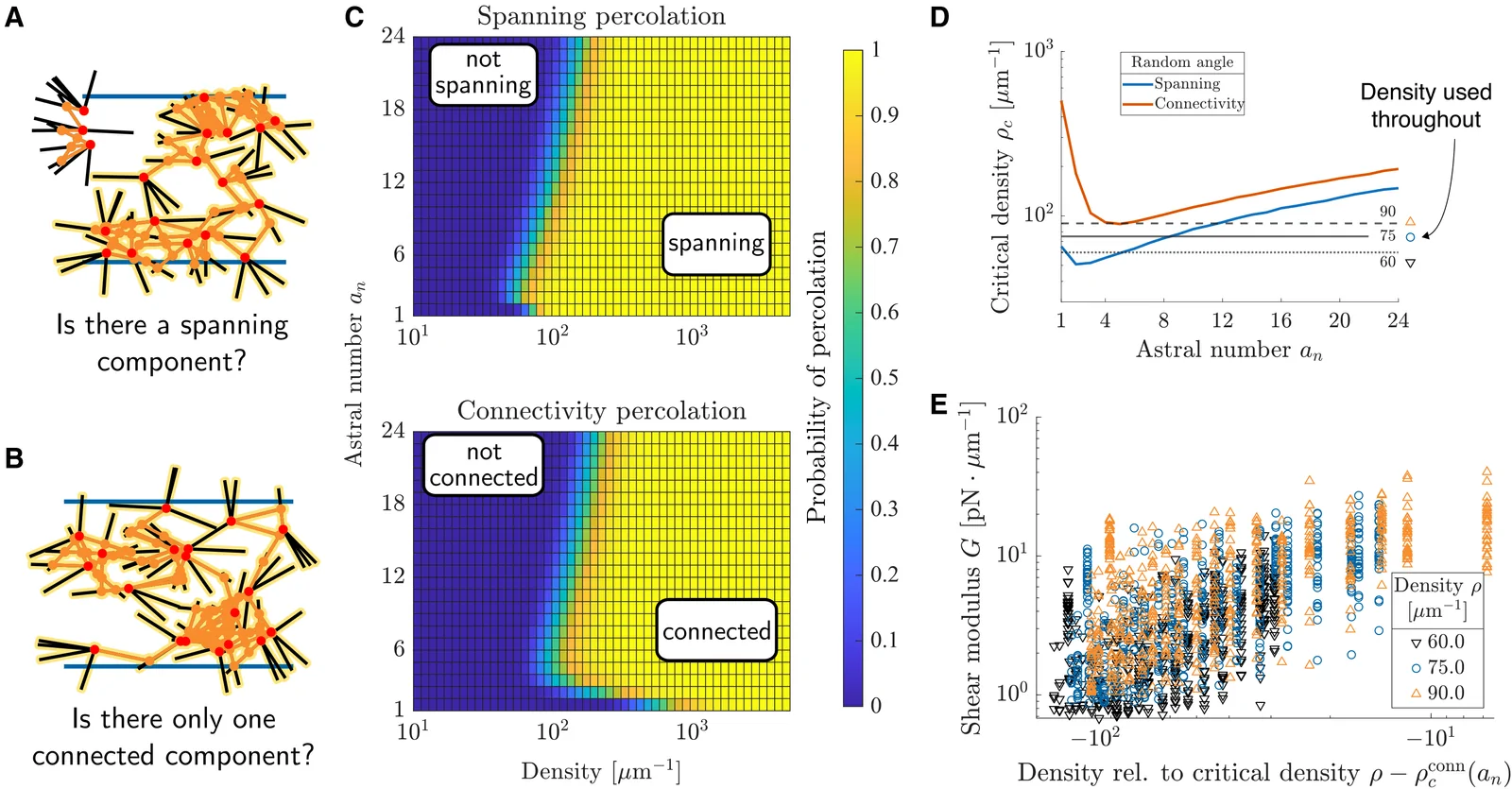
Mechanical rigidity is associated with proximity to percolation thresholds. (*a*) Schematic of a connected component that spans between the top and bottom edges of the domain. (*b*) Schematic of a network where all asters are connected into a single (unique) component. (*c*) Heatmaps showing percolation probabilities for astral networks with *ℓ* = 0.1 μm and *s* = 1 μm. Probabilities were estimated from *N* = 2000 networks at each sampled density and astral number. (*d*) Critical percolation densities estimated from the data in (*c*) as a function of astral number. The critical percolation density is defined as the filament density where the percolation probability reaches 0.5. Horizontal reference lines show the network densities present in (*e*). Note that *ρ* = 75 μm^−1^ was used in Figs. 2, 3, and 4. (*e*) Shear modulus versus $\Delta \rho =\rho -\rho_{c}^{\text{conn}}\left(a_{n}\right)$, the difference between the filament density in the simulated network *ρ* and the critical connectivity percolation density $\rho_{c}^{\text{conn}}\left(a_{n}\right)$ from (*d*). Three network densities are shown (see also Fig. S4*a*).

We computed two different percolation probabilities for finite-size astral networks (*ℓ* = 0.1 μm, *s* = 1 μm) via Monte Carlo sampling (see materials and methods for details). Given the mode of external stress we are interested in, we define two distinct notions of connectedness and therefore percolation. First, we investigated the appearance of “spanning” components in astral networks, defined as a set of connected asters that contains a cross-link both above and below the two system boundaries (Fig. 5 *a*). The existence of a spanning component is sufficient for an astral network to withstand applied force. Second, we investigated the emergence of a “unique” connected component (Fig. 5 *b*), i.e., when all the asters in a network cross-link into a single bulk material. We refer to this percolation mode as “connectivity” percolation. The computed percolation probabilities are shown in Fig. 5 *c*. As expected, for either definition, the probability transitions rapidly, and there is only a small region of parameters with subpopulations of both connected and unconnected networks.

Notably, the percolation thresholds appear to be nonmonotonic in astral number. We estimated these thresholds via computing the density where the percolation probability reaches 0.5, henceforth referred to as the critical densities $\rho_{c}^{\text{span}}\left(a_{n}\right)$ or $\rho_{c}^{\text{conn}}\left(a_{n}\right)$. (Note that these quantities are approximations for the infinite-extent percolation threshold as *s* → *∞* as shown in Fig. S8.) These critical densities are plotted in Fig. 5 *d*. Although both curves exhibit a minimum at low but nonzero astral number, we observed that the region of optimality in the connectivity threshold curve corresponds to the region of optimality in Fig. 2 *b*. We thus speculated that the proximity of the network density to the critical connectivity density was a predictor of network rigidity. To test this, we aggregated shear modulus samples from three different network densities *ρ* and various *a*_*n*_ (see also Fig. S4) and plotted shear modulus against the distance to critical density $\rho -\rho_{c}^{\text{conn}}\left(a_{n}\right)$ (Fig. 5 *e*). We see a clear correlation between rigidity and network density relative to $\rho_{c}^{\text{conn}}$ across astral number and network density (Pearson correlation of log-scale data 0.6352). This suggests that the dependence of rigidity on astral number may be due in part to the proximity of the density to the critical connectivity density.

Specifically, the relationship we observe is with the critical density for “connectivity” percolation. In contrast to the “spanning” definition of percolation, connectivity is neither required nor sufficient for a network to withstand applied force. This is a wider conclusion than the binary property of whether a particular network is percolated or not in the spanning sense, since the relationship we identify is applicable even when comparing, e.g., two networks both on one side of the connectivity percolation threshold.

**Figure 6:**
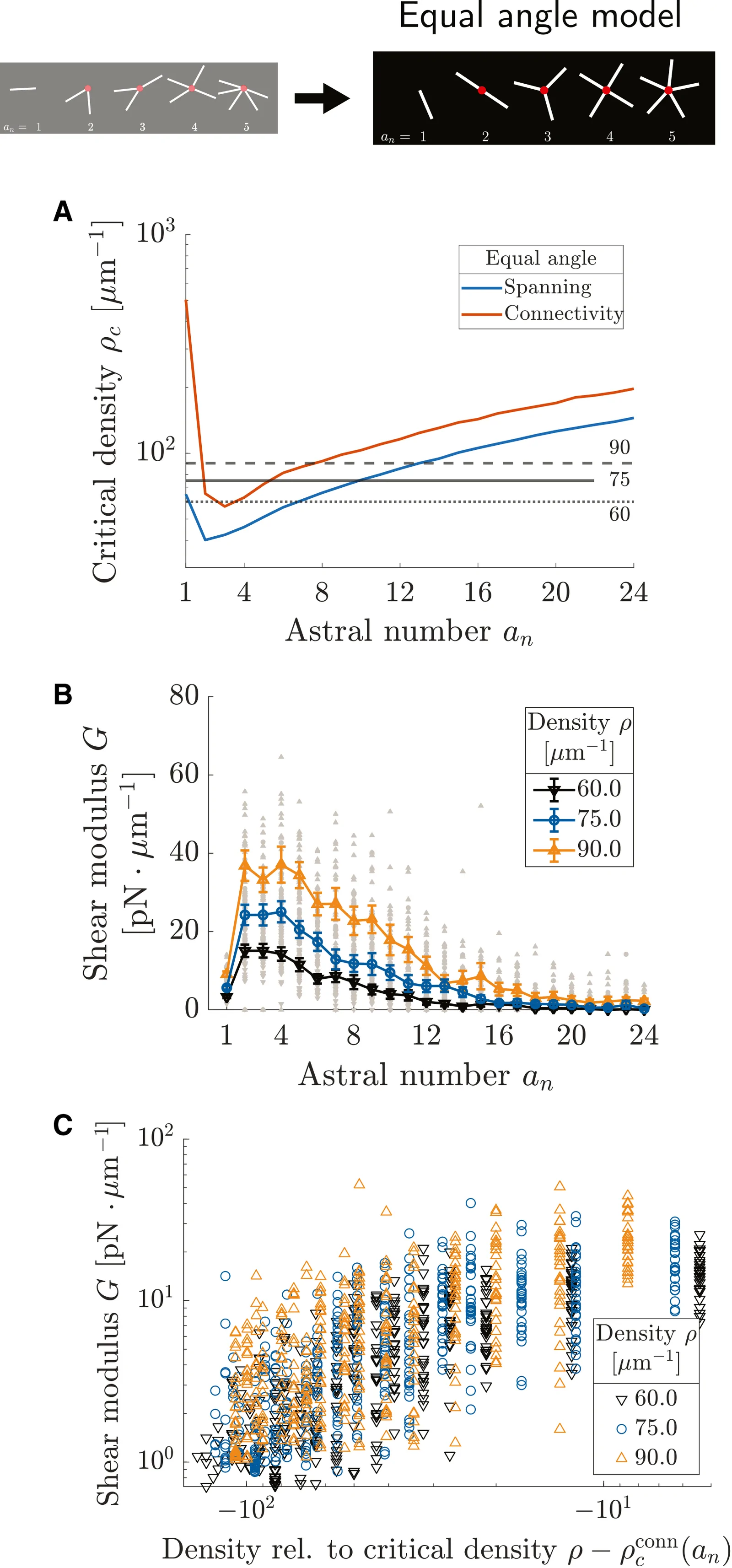
Shifting percolation threshold with model modification to equal angle asters. (*a*) Critical percolation densities for equal angle asters, estimated with the same procedure as in Fig. 5. Horizontal reference lines show the network densities present in (*c*). (*b*) Shear modulus versus astral number for equal angle asters. (*c*) Shear modulus versus $\Delta \rho =\rho -\rho_{c}^{\text{conn}}\left(a_{n}\right)$ for equal angle asters. Values of $\rho_{c}^{\text{conn}}\left(a_{n}\right)$ are from (*a*). Three network densities are shown (see also Fig. S4*a*).

If the above reasoning is sound, then a model modification that alters the percolation threshold would be expected to alter the optimal astral number. One model modification that might do so is to assume asters have filaments with equal angular spacing about the astral center, so that, e.g., *a*_*n*_ = 6 would have filaments separated by 60°. In Fig. 6 *a*, we confirm that this indeed alters the percolation threshold (connectivity occurs at a lower density, as expected). In Fig. 6 *b*, *c*, and S4, we perform mechanical measurements of these networks, and confirm that 1) the optimum is altered, and 2) the relationship between proximity-to-percolation threshold and mechanical strength remains. Moreover, the scatter plots for random and equal angle asters collapse onto each other (Fig. S4 *c*). Taken together, this provides strong evidence that the proximity to the percolation threshold determines in part the mechanical rigidity of astral structures.

## Discussion

In a seminal work, Wilhelm et al. (6) studied a simple model with only two molecular components: semiflexible filaments and interfilament cross-linkers, in two dimensions. They introduced the name “Mikado model,” in reference to a children’s game with similar architecture, and found nontrivial behavior including a new universality class of mechanical response. Continuing this agenda, we study a simple model with three molecular components: inextensible filaments, mechanical linkers creating astral centers, and mechanical linkers creating interaster cross-links, thus creating an “astral Mikado model.” In both cases, the simplicity allows the dissection of the specific role of model features, for example by disentangling the cause of astral architectures with the consequence of astral architecture, and by disentangling bulk (passive) stress response from active contractility. The simplicity also allows efficient simulation of large systems over many parameters, which would be computationally expensive in models with more features (35,36).

The major finding of this work is an optimal astral number that maximizes mechanical strength, both shear modulus and Young’s modulus, for a fixed density of F-actin filaments. As other parameters are varied, like density of F-actin, the parameter dependence of mechanical moduli is nonlinear and nonmonotonic, but we find the dependency largely aligns with a single metric, the density difference with the percolation threshold density (Fig. 5 *e*). This suggests that the strength of the network is a consequence of the heterogeneity in local filament density, which creates gaps in the network, rather than the constraints of different numbers of filaments and joints as in classical Maxwell counting (33).

In terms of problem-solving strategies selected by cells, a maximum rigidity may be desirable in some cases for cell integrity (37), and undesirable in other cases, for example during cell migration through a fine mesh (38,39). So, how does the astral number of in vivo F-actin networks compare with the optimal number we found here? Xia et al. (17) used super-resolution microscopy to study the F-actin cortex in embryonic stem cells. They identified astral centers (excluding interastral crossovers by filtering by the molecular constituents of astral centers) and then counted the filaments emanating from these. They found *a*_*n*_ ≈ 4 with an interquartile range of 3–5, in remarkable agreement with the optimum identified by the astral Mikado model (Fig. 2). This astral number is also roughly consistent with images of (15) and quantification in (21), in different cellular contexts. Thus, this may provide an example where a single biophysical parameter optimizes a clearly defined objective function in a cell biological system. Indeed, synthetic aster-based materials have been studied as a possible meta-material (40,41,42).

Recently, Garcia-Arcos et al. (27) demonstrated that migrating cells locally and dynamically tune their mechanical properties to navigate complex environment. The authors inferred a phase diagram depending on F-actin density and myosin contractility (their Fig. 5 *e*). Although we do not explicitly consider myosin contractility, Wollrab et al. (28) demonstrated both in vivo and in silico that myosin organizes F-actin into astral structures, in agreement with other work (29). Thus, if we assume that myosin activity causally induces astral structure, then the vertical axis of the phase diagram we show in Fig. 5 *c* roughly corresponds to myosin contractility. With this correspondence, the phase diagram in Fig. 5 *c* is remarkably similar to the phase diagram inferred by (27). This suggests that dynamic astral nanoscale architecture may provide the knob allowing cells to dynamically tune their mechanics.

Future models of astral cytoskeletal networks will explore additional features present in vivo. One example is polydispersity in astral number and in filament length. Since many distributions can correspond to the same mean astral number, it would be interesting to examine how properties of the distribution (e.g., number of aster types, variance) modify the trends seen here. Regarding filament length, previous investigations (32) demonstrated a role for filament length heterogeneity. The rigidity of such networks appears to be sensitive to the presence of long filaments both experimentally (17) and computationally (32), and modeling can explore how astral nanostructure magnifies (or diminishes) this sensitivity. Another example is cross-linker turnover, i.e., dynamic binding and unbinding. The turnover of cross-linkers has been shown to play a major role in several cytoskeletal structures, including the cytokinetic ring (13) and the cortex during endocytosis (43). The dynamics of cross-linkers in astral networks could give rise to nontrivial aster-dependent dynamics, i.e., viscoelastic mechanical properties.

## Data and code availability

• All code has been deposited on Github and Zenodo. The fork of Cytosim is at Zenodo: https://doi.org/10.5281/zenodo.18970832.
• The configurations, analysis and geometry sampling and analysis are at Zenodo: https://doi.org/10.5281/zenodo.18970060.

## Acknowledgments

We thank Shannon McFadden and Andrew Rusli for useful discussion. This work was supported by 10.13039/100000001NSF DMS 2052668 and 10.13039/100000002NIH T32 GM136624.

## Author contributions

B.B. designed research, developed geometric analysis software, performed all simulations, analyzed results, and wrote the paper. J.A. designed research and wrote the paper.

## Declaration of interests

The authors declare no competing interests.

## Supporting material

Document S1. Figures S1–S9

Document S2. Article plus supporting material
