# Supplementary material for: Astral architecture can enhance mechanical strength of cytoskeletal networks by modulating percolation thresholds
Source: Biophys J. 2026 Mar 31;125(8):1958–67. doi: 10.1016/j.bpj.2026.03.028 (PMC13351913; doi:10.1016/j.bpj.2026.03.028)
Supplement: Document S1. Figures S1–S9 [file mmc1.pdf]

Biophysical Journal, Volume *125*

Supplemental information

Astral architecture can enhance mechanical strength of cytoskeletal networks by modulating percolation thresholds

Brady Berg and Jun Allard

Supplemental material

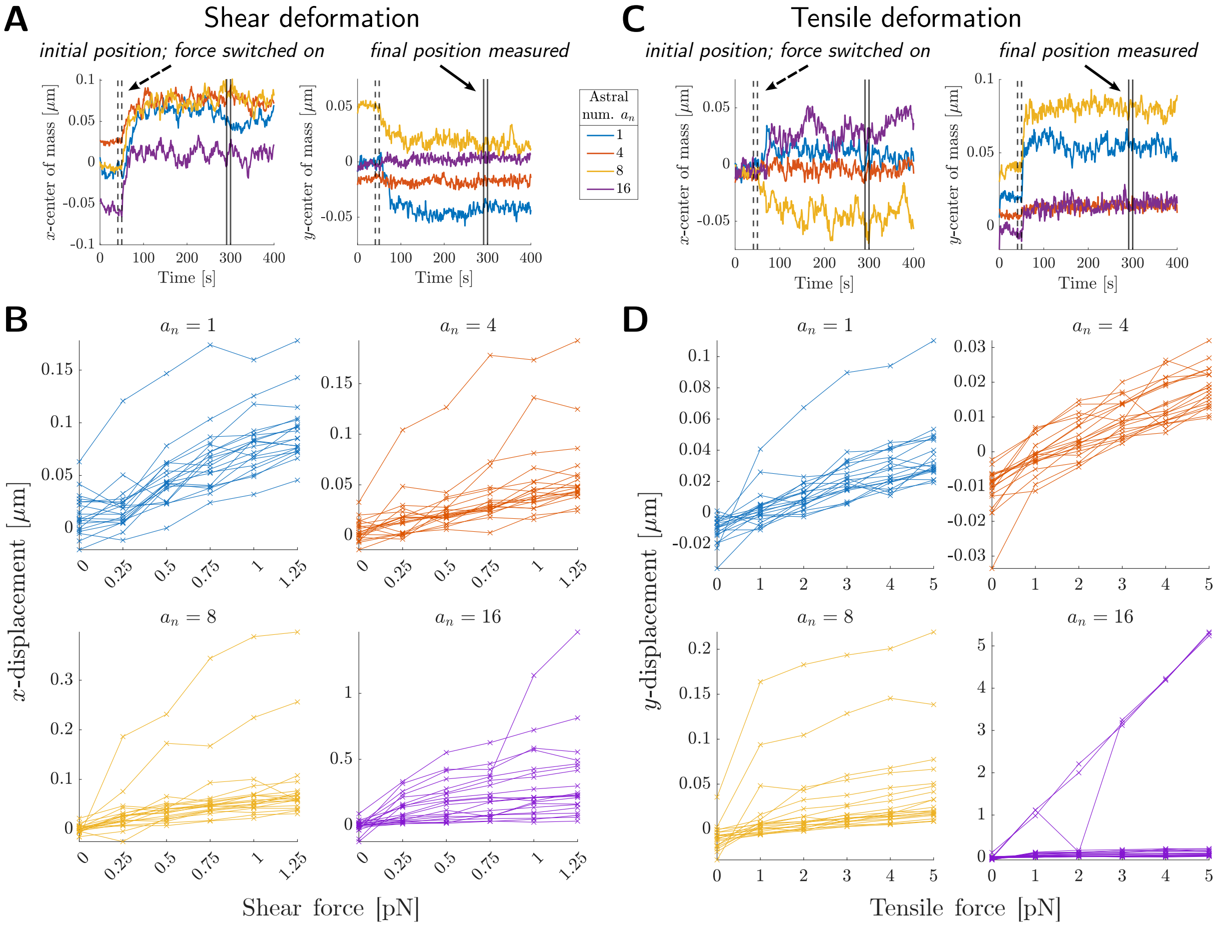

Figure S1: **Force-displacement measurements using Cytosim at physiological temperature.** (a) Center of mass coordinates over time for individual networks experiencing a shear force of $1.25\,\mathrm{pN}$ (in the positive x-direction). Initial position was measured in the interval indicated by dashed vertical lines, and final position was measured in the interval indicated by solid vertical lines. Force was switched on at the end of the initial position measurement window. Legend is common to (a) and (c). Movies of these networks are provided in S1, S2, S3, and S4. (b) Horizontal displacement data at selected astral numbers (subset of the data used to generate Figure S2b). (c) Center of mass coordinates over time for individual networks experiencing a tensile force of $5\,\mathrm{pN}$ (in the positive y-direction). Initial position was measured in the interval indicated by dashed vertical lines, and final position was measured in the interval indicated by solid vertical lines. Force was switched on at the end of the initial position measurement window. Movies of these networks are provided in S5, S6, S7, and S8. (d) Vertical displacement data at selected astral numbers (subset of the data used to generate Figure S2b). For (b) and (d), $N = 20$ networks were simulated per astral number.

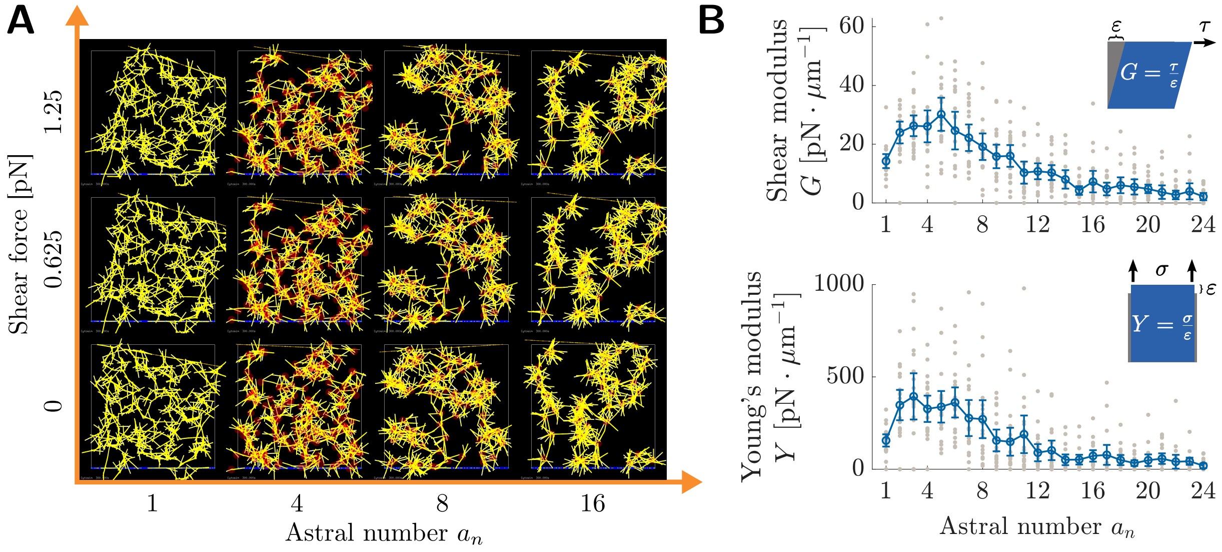

Figure S2: **Rigidity of astral filamentous networks exhibits a maximum at intermediate astral number, even at fixed density (physiological temperature).** (a) Steady-state snapshots of astral network deformation in response to applied shear forces. All tiles have equal total number of filaments. (b) Elastic moduli of astral networks at density $\rho = 75\,\mu\mathrm{m}^{-1}$ as a function of astral number. Upper: shear modulus, lower: Young's (tensile) modulus. Plots show mean and 95% CIs from $N = 20$ network samples (see Materials and Methods for details). Individual network moduli are shown (gray markers).

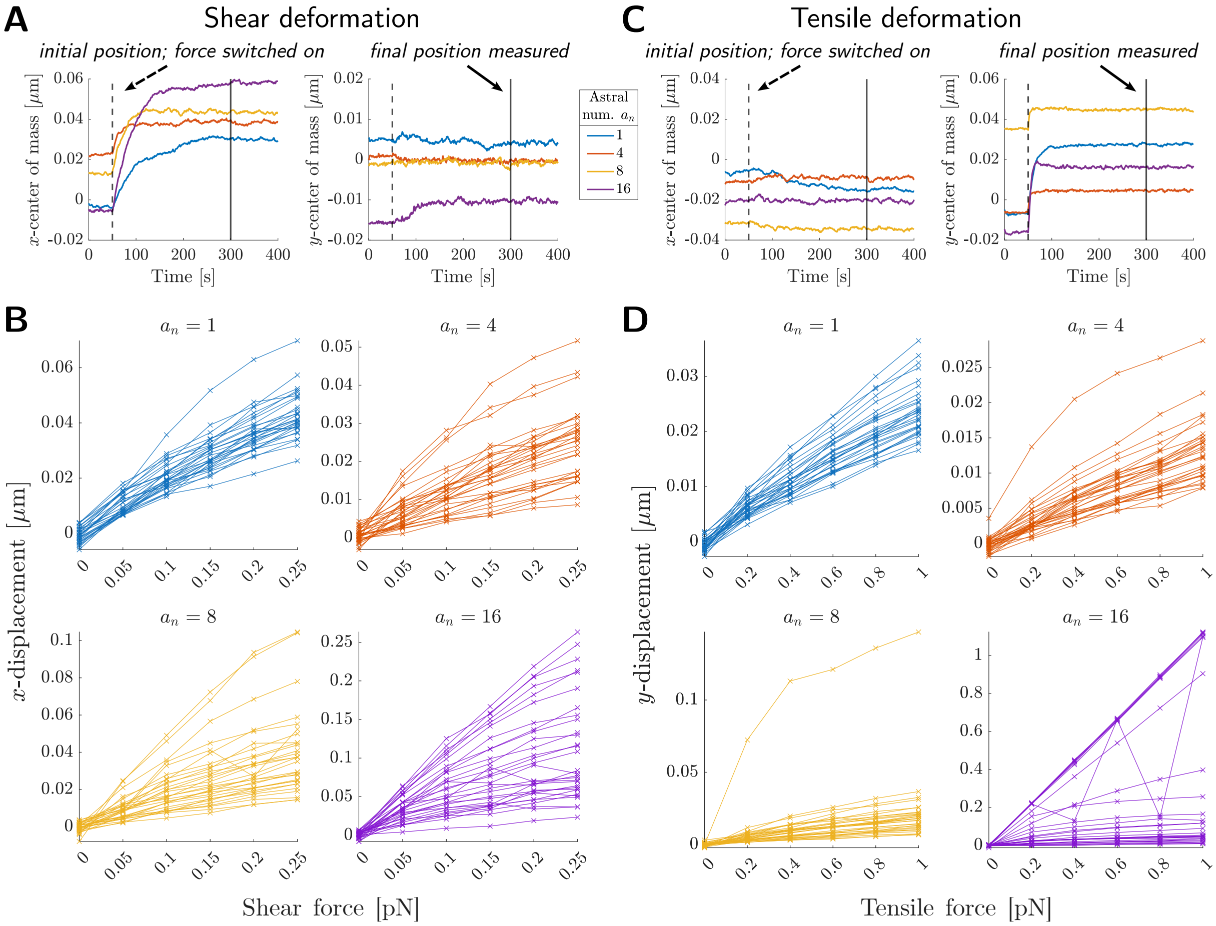

Figure S3: **Force-displacement measurements using Cytosim (reduced k_BT parameters).** (a) Center of mass coordinates over time for individual networks experiencing a shear force of 0.25 pN (in the positive x-direction). Dashed vertical line indicates when the force was switched on, and solid vertical line indicates when final network positions were recorded. Legend is common to (a) and (c). Movies of these networks are provided. in S9, S10, S11, and S12. (b) Horizontal displacement data at selected astral numbers (subset of the data used to generate Figure 2b). (c) Center of mass coordinates over time for individual networks experiencing a tensile force of 1 pN (in the positive y-direction). Dashed vertical line indicates when the force was switched on, and solid vertical line indicates when final network positions were recorded. Movies of these networks are provided in S13, S14, S15, and S16. (d) Vertical displacement data at selected astral numbers (subset of the data used to generate Figure 2b). For (b) and (d), $N = 30$ networks were simulated per astral number.

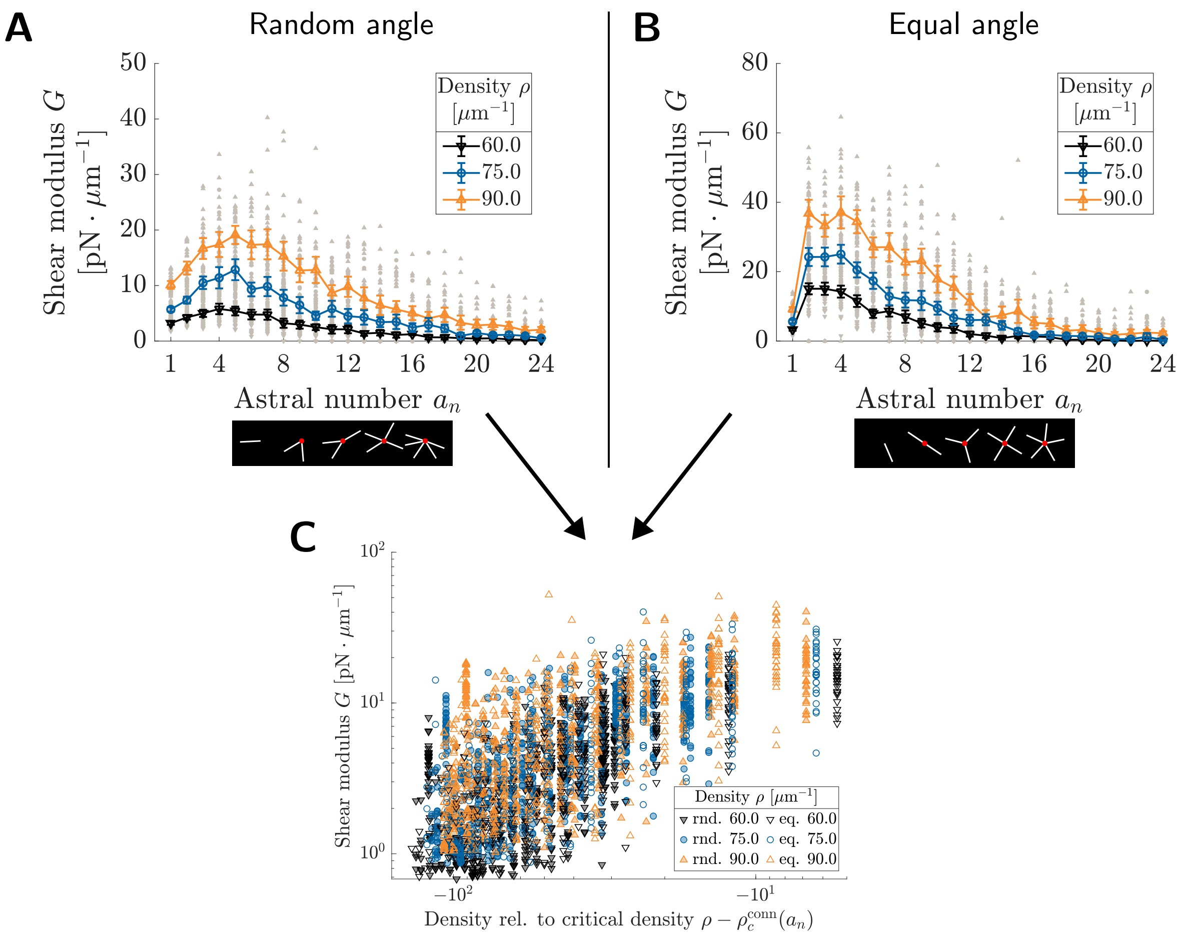

Figure S4: **Rigidity peak is insensitive to network density, and sensitive to geometry modification.** (a) Shear modulus as a function of astral number for three filament densities ρ. The location of the rigidity peak is insensitive to filament density. Astral filaments are oriented uniformly at random. (b) Model modification in which astral filaments have equal angular spacing. Shear modulus as a function of astral number for three filament densities ρ. (c) Shear modulus versus $\Delta\rho = \rho - \rho_c^{\text{conn}}(a_n)$ showing both random angle asters and equal angle asters (i.e., a superposition of Figure 5e and Figure 6c). In (a) and (b), plots show mean and 95% CIs, along with individual network moduli (gray markers). The $\rho = 75\,\mu\text{m}^{-1}$ curve in (a) is reproduced from Figure 2b, where $N = 30$ networks were sampled per astral number. The remaining curves were also generated using $N = 30$ network samples per astral number.

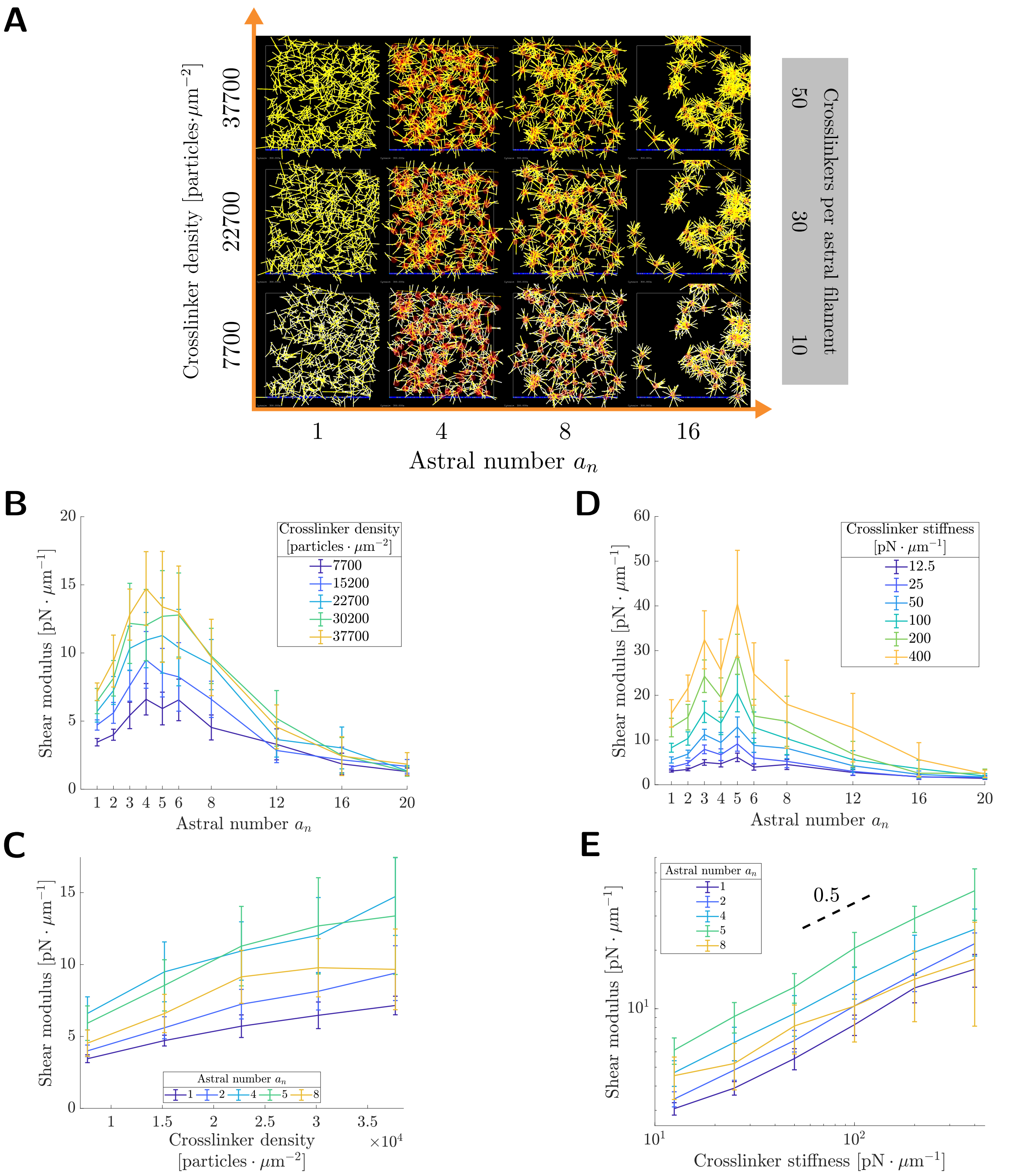

Figure S5: **Crosslinker properties modulate rigidity magnitudes but not optimality trend.** (a) Steady-state snapshots of astral network deformation at various crosslinker densities. Crosslinker particles are shown in yellow, and astral centers are marked in red. All networks have filament density $\rho = 75\,\mu\text{m}^{-1}$ and experience a shear force of magnitude $0.25\,\text{pN}$. (b) Shear modulus as a function of astral number for a series of crosslinker densities. (c) Shear modulus as a function of crosslinker density for a series of astral numbers. (d) Shear modulus as a function of astral number for a series of crosslinker stiffnesses. (e) Shear modulus as a function of crosslinker stiffness for a series of astral numbers. Note that "stiffness" refers to the linear stretch resistance of each crosslinker, and crosslinkers do not individually exert torques. Plots show mean and 95% CIs computed from $N = 15$ networks per astral number. See Table 1 for default parameter values used outside of this Figure.

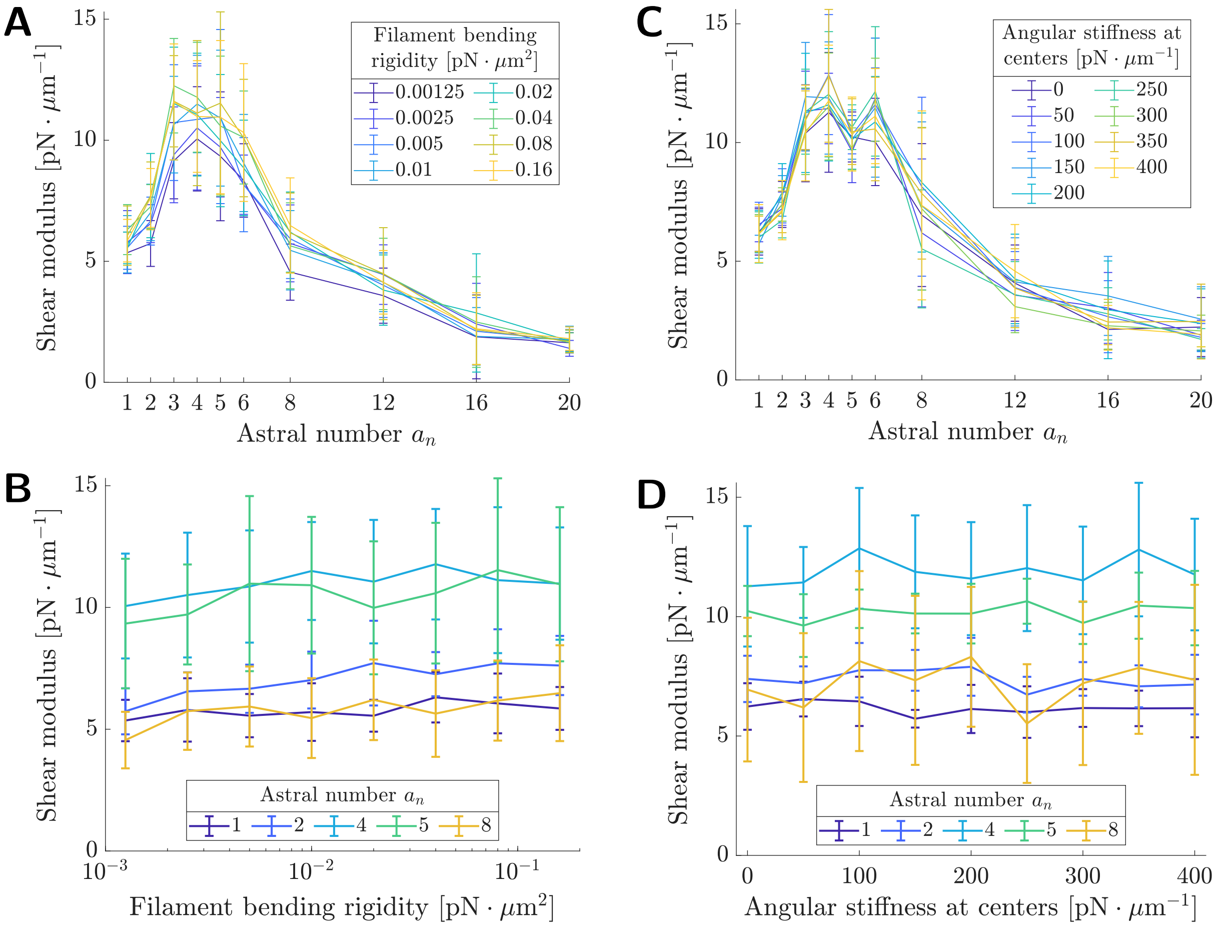

Figure S6: **Peak in rigidity is only weakly sensitive to bending of filaments and angular stiffness at astral centers.** (a) Shear modulus as a function of astral number for a series of filament bending rigidities. (b) Shear modulus as a function of filament bending rigidity for a series of astral numbers. (c) Shear modulus as a function of astral number for a series of angular stiffnesses at astral centers. (d) Shear modulus as a function of angular stiffness at astral centers for a series of astral numbers. Plots show mean and 95% CIs computed from $N = 10$ networks per astral number. See Table 1 for default parameter values used outside of this Figure.

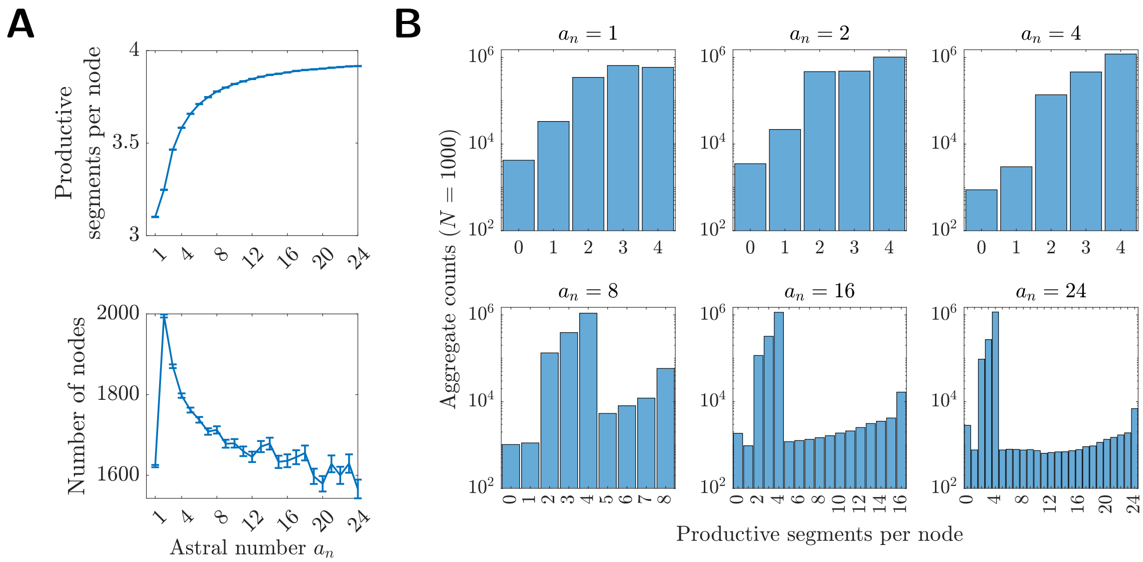

Figure S7: **Number of segments per node, including both astral centers and inter-aster crosslinks.** (a) Upper: Number of productive (i.e. non-dangling) segments per node as a function of astral number. Lower: Number of nodes (crosslinks) in astral networks as a function of astral number. Data shown are mean and 95% CIs estimated from 1000 networks per astral number. (b) Nodes from 1000 astral networks sorted by the number of productive segments connected to each node.

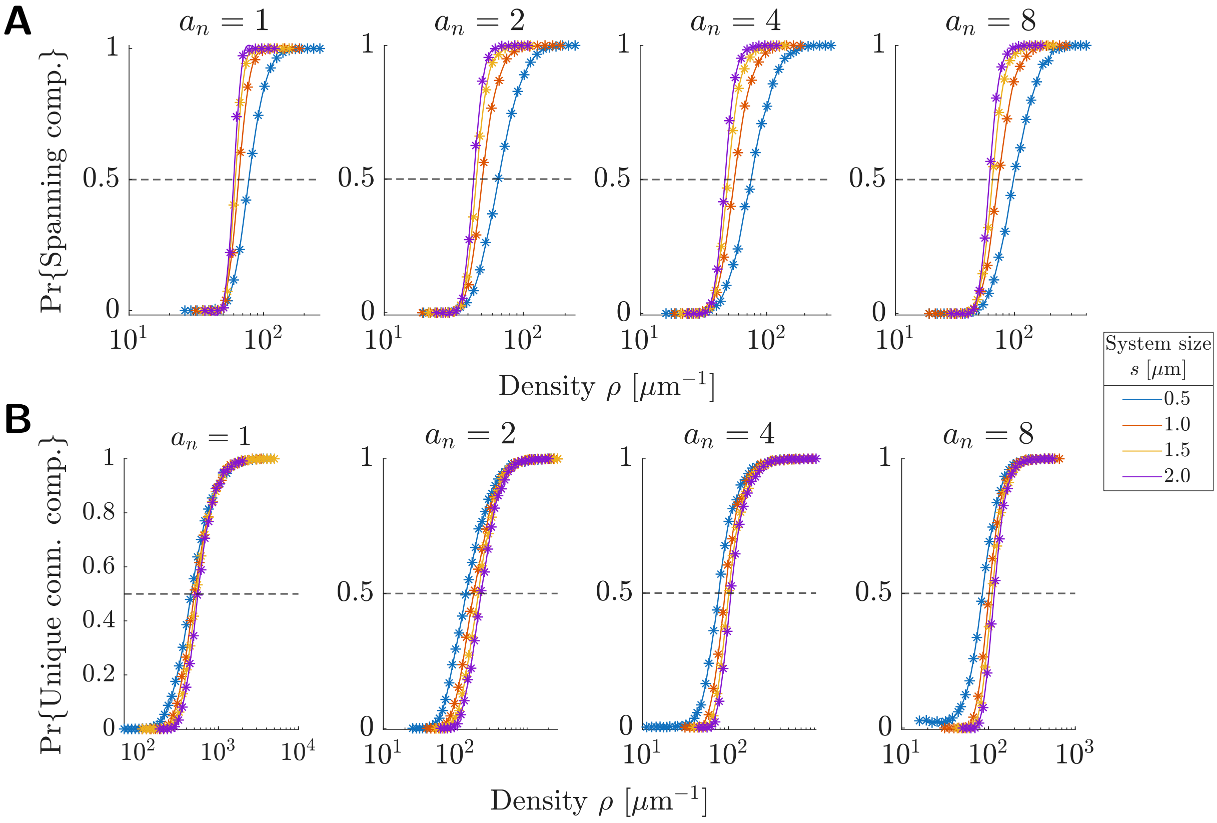

Figure S8: **Data and fits used to estimate critical percolation densities.** (a) Probability that a network contains a spanning component as a function of network density ρ. (b) Probability that a network contains a unique connected component as a function of network density ρ. Markers show percolation probabilities estimated from $N = 2000$ networks; curves show smoothing spline fits used to estimate critical percolation densities (intersection with $p = 0.5$). Data shown for selected astral numbers a_n and a family of system sizes s. All network filaments have length $\ell = 0.1\,\mu$m. Only data for $s = 1\,\mu$m were used to generate Figure 5d.

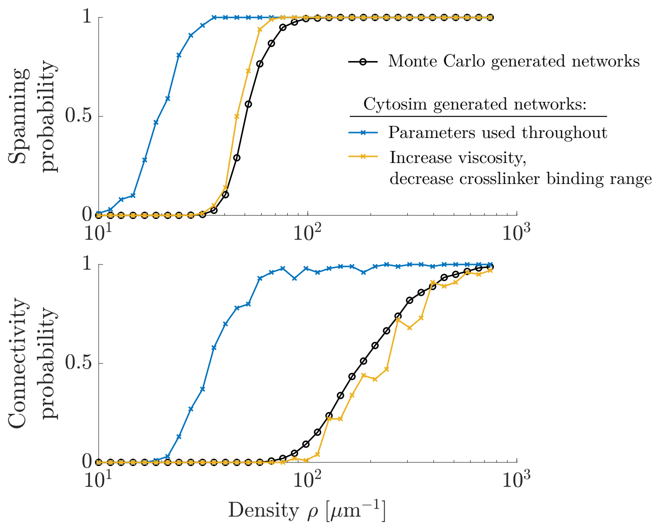

Figure S9: **Comparison of stochastic molecular dynamics networks from Cytosim and ideal geometry networks from Monte Carlo generation.** Black open circles: percolation probabilities computed from Monte Carlo generated networks, where connectivity is defined by whether linear filaments overlap. Blue x: using crosslinker connections reported by Cytosim at simulation parameters used in Main Text and reported in Table 1 and 2. Yellow x: using crosslinker connections reported by Cytosim, at increased viscosity 2000-fold to 10^3pN/µm^2 and reduced crosslinker binding range 10^{-4}µm.

Supplemental Videos

Shear force (physiological temperature)

Video S1: Simulation of a filament network with astral number $a_n = 1$ (i.e., a classical "Mikado" network) experiencing a shear force at physiological temperature. For times $0 \leq t < 50\,\text{sec}$, the network crosslinks into its initial position while no external force is applied. At time $t = 50\,\text{sec}$, a constant shear force of magnitude $1.25\,\text{pN}$ is switched on and maintained for the remainder of the simulation.

Video S2: Simulation of an astral filament network with astral number $a_n = 4$ experiencing a shear force at physiological temperature. For times $0 \leq t < 50\,\text{sec}$, the network crosslinks into its initial position while no external force is applied. At time $t = 50\,\text{sec}$, a constant shear force of magnitude $1.25\,\text{pN}$ is switched on and maintained for the remainder of the simulation.

Video S3: Simulation of an astral filament network with astral number $a_n = 8$ experiencing a shear force at physiological temperature. For times $0 \leq t < 50\,\text{sec}$, the network crosslinks into its initial position while no external force is applied. At time $t = 50\,\text{sec}$, a constant shear force of magnitude $1.25\,\text{pN}$ is switched on and maintained for the remainder of the simulation.

Video S4: Simulation of an astral filament network with astral number $a_n = 16$ experiencing a shear force at physiological temperature. For times $0 \leq t < 50\,\text{sec}$, the network crosslinks into its initial position while no external force is applied. At time $t = 50\,\text{sec}$, a constant shear force of magnitude $1.25\,\text{pN}$ is switched on and maintained for the remainder of the simulation.

Extensional force (physiological temperature)

Video S5: Simulation of a filament network with astral number $a_n = 1$ (i.e., a classical "Mikado" network) experiencing a tensile force at physiological temperature. For times $0 \leq t < 50\,\text{sec}$, the network crosslinks into its initial position while no external force is applied. At time $t = 50\,\text{sec}$, a constant tensile force of magnitude $5\,\text{pN}$ is switched on and maintained for the remainder of the simulation.

Video S6: Simulation of an astral filament network with astral number $a_n = 4$ experiencing a tensile force at physiological temperature. For times $0 \leq t < 50\,\text{sec}$, the network crosslinks into its initial position while no external force is applied. At time $t = 50\,\text{sec}$, a constant tensile force of magnitude $5\,\text{pN}$ is switched on and maintained for the remainder of the simulation.

Video S7: Simulation of an astral filament network with astral number $a_n = 8$ experiencing a tensile force at physiological temperature. For times $0 \leq t < 50\,\text{sec}$, the network crosslinks into its initial position while no external force is applied. At time $t = 50\,\text{sec}$, a constant tensile force of magnitude $5\,\text{pN}$ is switched on and maintained for the remainder of the simulation.

Video S8: Simulation of an astral filament network with astral number $a_n = 16$ experiencing a tensile force at physiological temperature. For times $0 \leq t < 50\,\text{sec}$, the network crosslinks into its initial position while no external force is applied. At time $t = 50\,\text{sec}$, a constant tensile force of magnitude $5\,\text{pN}$ is switched on and maintained for the remainder of the simulation.

Shear force (reduced k_BT)

Video S9: Simulation of a filament network with astral number $a_n = 1$ (i.e., a classical "Mikado" network) experiencing a shear force (reduced k_BT parameters). For times $0 \leq t < 50\,\mathrm{sec}$, the network crosslinks into its initial position while no external force is applied. At time $t = 50\,\mathrm{sec}$, a constant shear force of magnitude $0.25\,\mathrm{pN}$ is switched on and maintained for the remainder of the simulation.

Video S10: Simulation of an astral filament network with astral number $a_n = 4$ experiencing a shear force (reduced k_BT parameters). For times $0 \leq t < 50\,\mathrm{sec}$, the network crosslinks into its initial position while no external force is applied. At time $t = 50\,\mathrm{sec}$, a constant shear force of magnitude $0.25\,\mathrm{pN}$ is switched on and maintained for the remainder of the simulation.

Video S11: Simulation of an astral filament network with astral number $a_n = 8$ experiencing a shear force (reduced k_BT parameters). For times $0 \leq t < 50\,\mathrm{sec}$, the network crosslinks into its initial position while no external force is applied. At time $t = 50\,\mathrm{sec}$, a constant shear force of magnitude $0.25\,\mathrm{pN}$ is switched on and maintained for the remainder of the simulation.

Video S12: Simulation of an astral filament network with astral number $a_n = 16$ experiencing a shear force (reduced k_BT parameters). For times $0 \leq t < 50\,\mathrm{sec}$, the network crosslinks into its initial position while no external force is applied. At time $t = 50\,\mathrm{sec}$, a constant shear force of magnitude $0.25\,\mathrm{pN}$ is switched on and maintained for the remainder of the simulation.

Extensional force (reduced k_BT)

Video S13: Simulation of a filament network with astral number $a_n = 1$ (i.e., a classical "Mikado" network) experiencing a tensile force (reduced k_BT parameters). For times $0 \leq t < 50\,\mathrm{sec}$, the network crosslinks into its initial position while no external force is applied. At time $t = 50\,\mathrm{sec}$, a constant tensile force of magnitude $1\,\mathrm{pN}$ is switched on and maintained for the remainder of the simulation.

Video S14: Simulation of an astral filament network with astral number $a_n = 4$ experiencing a tensile force (reduced k_BT parameters). For times $0 \leq t < 50\,\mathrm{sec}$, the network crosslinks into its initial position while no external force is applied. At time $t = 50\,\mathrm{sec}$, a constant tensile force of magnitude $1\,\mathrm{pN}$ is switched on and maintained for the remainder of the simulation.

Video S15: Simulation of an astral filament network with astral number $a_n = 8$ experiencing a tensile force (reduced k_BT parameters). For times $0 \leq t < 50\,\mathrm{sec}$, the network crosslinks into its initial position while no external force is applied. At time $t = 50\,\mathrm{sec}$, a constant tensile force of magnitude $1\,\mathrm{pN}$ is switched on and maintained for the remainder of the simulation.

Video S16: Simulation of an astral filament network with astral number $a_n = 16$ experiencing a tensile force (reduced k_BT parameters). For times $0 \leq t < 50\,\mathrm{sec}$, the network crosslinks into its initial position while no external force is applied. At time $t = 50\,\mathrm{sec}$, a constant tensile force of magnitude $1\,\mathrm{pN}$ is switched on and maintained for the remainder of the simulation.
